# Mint leaves: Dried, fresh, and spoiled dataset for condition analysis and machine learning applications

**DOI:** 10.1016/j.dib.2023.109717

**Published:** 2023-10-24

**Authors:** Rohini Jadhav, Yogesh Suryawanshi, Yashashree Bedmutha, Kailas Patil, Prawit Chumchu

**Affiliations:** a Bharati Vidyapeeth College of Engineering, Pune, India; b Vishwakarma University, Pune, India; c Kasetsart University, Sriracha, Thailand

**Keywords:** Condition analysis, Image classification, Machine learning, Mint leaves, Dataset, Quality assessment

## Abstract

We present a comprehensive dataset of 5,323 images of mint (pudina) leaves in various conditions, including dried, fresh, and spoiled. The dataset is designed to facilitate research in the domain of condition analysis and machine learning applications for leaf quality assessment. Each category of the dataset contains a diverse range of images captured under controlled conditions, ensuring variations in lighting, background, and leaf orientation. The dataset also includes manual annotations for each image, which categorize them into the respective conditions. This dataset has the potential to be used to train and evaluate machine learning algorithms and computer vision models for accurate discernment of the condition of mint leaves. This could enable rapid quality assessment and decision-making in various industries, such as agriculture, food preservation, and pharmaceuticals. We invite researchers to explore innovative approaches to advance the field of leaf quality assessment and contribute to the development of reliable automated systems using our dataset and its associated annotations.

Specifications Table

**Table:** 

Subject:	Applied Machine Learning, Agriculture
Specific subject area:	Agronomy & Crop Science
Data format:	Raw
Type of data:	Images
Description of data collection:	The dataset of 5,323 images of mint leaves was meticulously collected at Vishwakarma University, Pune. Leaves were sourced from local gardens and selected to represent three distinct conditions: dried, fresh, and spoiled. To ensure a diverse set of images, variations in lighting, background, and leaf positioning were intentionally incorporated during the image capture process. Each leaf was individually photographed under controlled conditions to accurately portray its condition. The captured images were saved in JPG format and resized to a resolution of 1280 × 768 pixels.
Data source location:	Vishwakarma University, Kondhwa Budruk, Maharashtra, Pune, India Longitude and Latitude: 18°27′37.8"N 73°53′00.9"E
Data accessibility:	Repository name: Pudina Leaf Dataset: Freshness Analysis Data identification number: 10.17632/nvbpydc3fs.1 Direct URL to data: https://data.mendeley.com/datasets/nvbpydc3fs/1

## 1 Value of the Data

• This mint leaves dataset has the potential to be used to address a variety of research questions in the field of leaf quality assessment such as how machine learning algorithms be used to accurately discern the condition of mint leaves?, can the dataset be used to automate the process of leaf quality assessment?, how can the dataset be used to improve the efficiency and accuracy of decision-making in the agriculture?
• The dataset provides a valuable resource for researchers and practitioners working on condition analysis, machine learning, and computer vision. It can be used to develop and evaluate algorithms for automated leaf quality assessment, contributing to advancements in these fields. Moreover, its potential applications in herbal medicine, agricultural studies, and culinary arts have been substantiated by recent studies [10], [11], [12], underscoring its significant value across diverse disciplines.
• Researchers can use this dataset as a benchmark for comparing the performance of different algorithms and methods for leaf quality assessment.
• The dataset can serve as an educational tool in computer science, machine learning, and image processing courses. Students can work with real-world data to gain practical experience in these fields.
• Machine Learning Applications: The availability of this dataset opens avenues for researchers to develop and compare advanced machine learning models for mint leaves quality detection and classification. It encourages innovative approaches and fosters collaborations in the field of plant pathology.

## 2 Data Description

The objective of the “Mint Leaves: Dried, Fresh, and Spoiled Dataset” is to serve as a comprehensive resource for advancing machine learning techniques in accurately discerning the condition of mint leaves. This dataset aims to facilitate the development, evaluation, and refinement of robust algorithms and models for precise classification. The dataset comprises a diverse collection of mint (pudina) leaf images, meticulously collected from various sources at Vishwakarma University, Pune. The dataset's primary objective is to provide a comprehensive resource for advancing research in condition analysis, particularly within the context of machine learning and computer vision applications for leaf quality assessment. Existing research works [[1], [2], [3], [4], [5]]] address specific image classification challenges and foster innovation in the field. Additionally, the 'Addressing misclassification in deep learning' paper [6] introduces the 'Merged Net' approach, which is relevant for improving classification accuracy in datasets, including the mint leaves dataset.

The primary objective of assembling this dataset is to facilitate the development, evaluation, and improvement of machine learning models and algorithms for accurately classifying the condition of mint leaves. By encompassing various conditions, including dried, fresh, and spoiled leaves, the dataset aims to enable researchers to create robust and effective solutions that can benefit industries such as agriculture, food preservation, and pharmaceuticals.

The dataset is composed of a total of 5,323 images, categorically distributed as follows:

• Dried Leaves: 1,881 images
• Fresh Leaves: 1,773 images
• Spoiled Leaves: 1,669 images

The distribution of images across these categories ensures a balanced representation of leaf conditions, thereby promoting the development of unbiased and accurate models.

The images within each category capture variations in lighting, background, and leaf orientation. This variability was intentionally incorporated to mimic real-world scenarios and challenges. Each image is manually annotated to indicate its specific condition category, providing ground truth labels for supervised learning tasks.

These images capture the leaves under varying light conditions, which is especially relevant for applications requiring robustness in different environments.

**Fig. 1:**
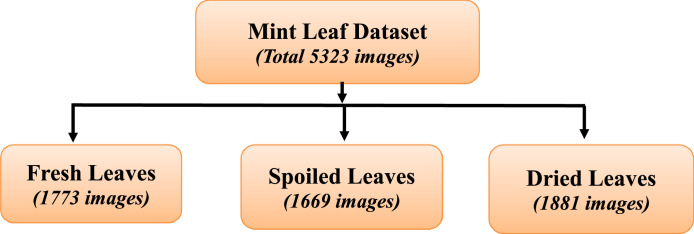
Directory structure of the mint leaves dataset.

Each category is labelled and organized in separate folders, ensuring easy access and identification of specific disease samples. Fig. 1 shows directory structure of the mint leaves dataset.

## 3 Experimental Design, Materials and Methods

### 3.1 Experimental design

**Fig. 2:**
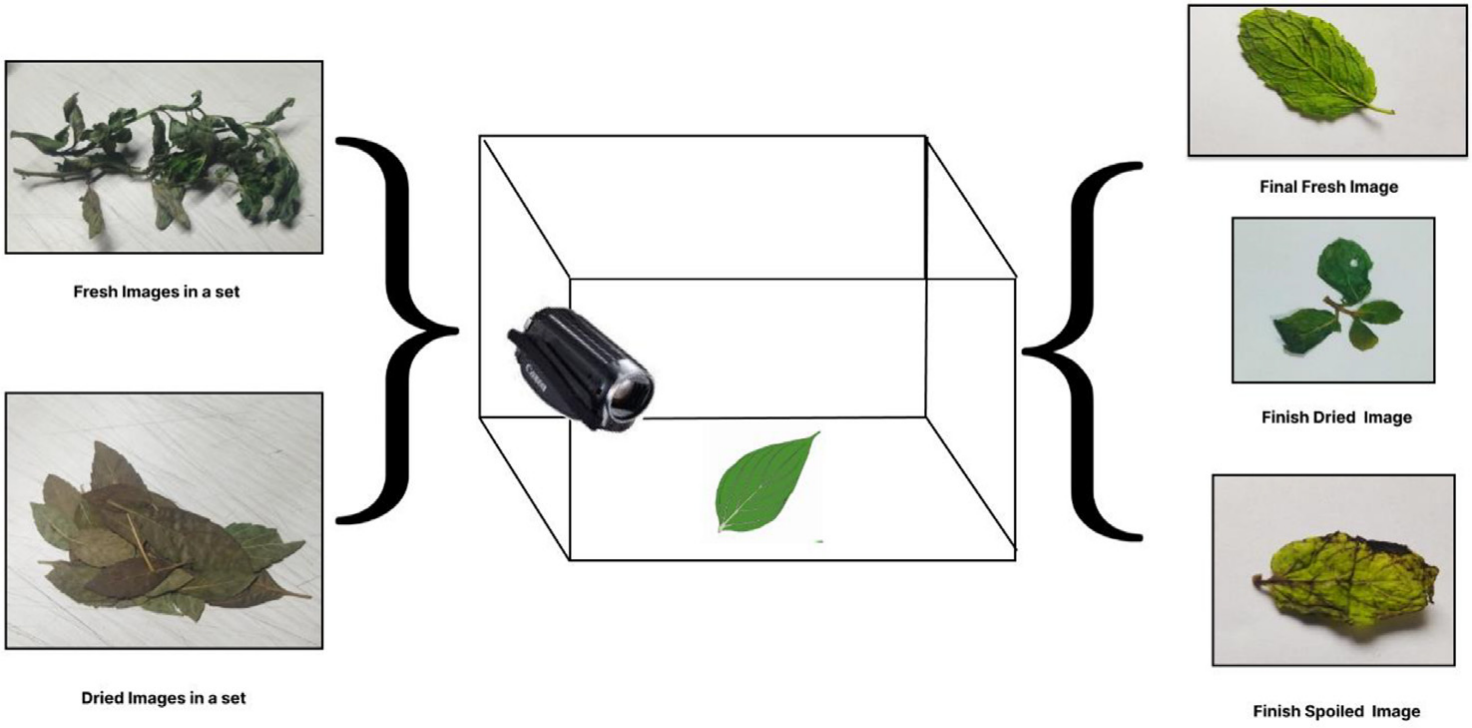
Experimental setup for mint leaves dataset.

**Fig. 3:**
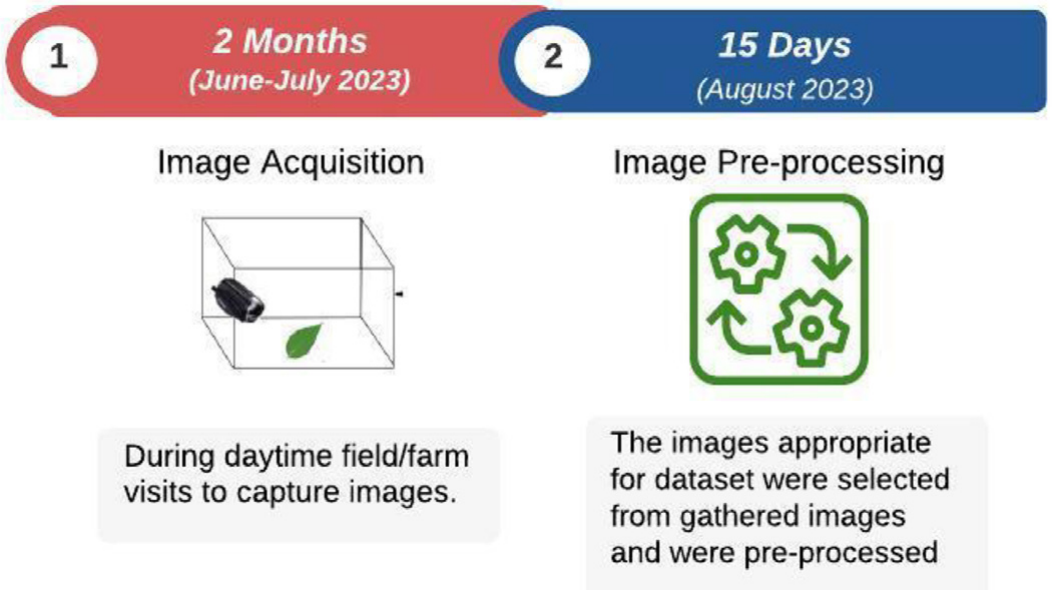
Data acquisition steps.

The images of mint leaves were captured using a Canon camcorder model HF R236, ensuring consistent image quality and resolution for each leaf specimen. Fig. 2 shows the experimental setup for dataset creation. The dataset encompasses three leaf conditions (dried, fresh, and spoiled), introducing variability in lighting and environmental factors to mimic real-world scenarios. The data acquisition process consisted of two main steps, as summarized in Fig. 3.

**Step 1: *Image Acquisition (Duration: April to July):*** During this phase, we conducted field visits in daylight to capture images related to various coconut tree diseases. The main goal was to create a comprehensive collection of disease-related images.
**Step 2: *Image Pre-processing (Duration: July):*** In this step, we used the Python script to improve photos of mint leaves by altering their brightness, saturation, and highlights.

Overall, the data acquisition process involved capturing images during field visits and subsequently preparing them through pre-processing to include in the dataset.

### 3.2 Materials or specification of image acquisition system

The camcorder used in the data acquisition process and the specifications of the captured images:

*LEGRIA HF R36 Camcorder:*

• Sensor Type HD CMOS, Size Diagonal: 340, 1/4.85 inch
• Focal Length 2.8 - 89.6 mm (35 mm Equivalent to 38.5 - 1232 mm)
• Aperture Range F1.8 - F4.5
• Aspect Ratio 16:09

The captured images were saved in JPG format and resized to a resolution of 1280 × 768 pixels. These specifications provide crucial details about the cameras used and the image properties obtained during the data acquisition process. Table 1 presents sample images of mint leaves dataset, providing a visual representation of the dataset used in this study.

**Table 1:** Sample images of mint leaves dataset.

Dried Mint Leaves	
Spoiled Mint Leaves	
Fresh Mint Leaves	

### 3.3 Method

**Fig. 4:**
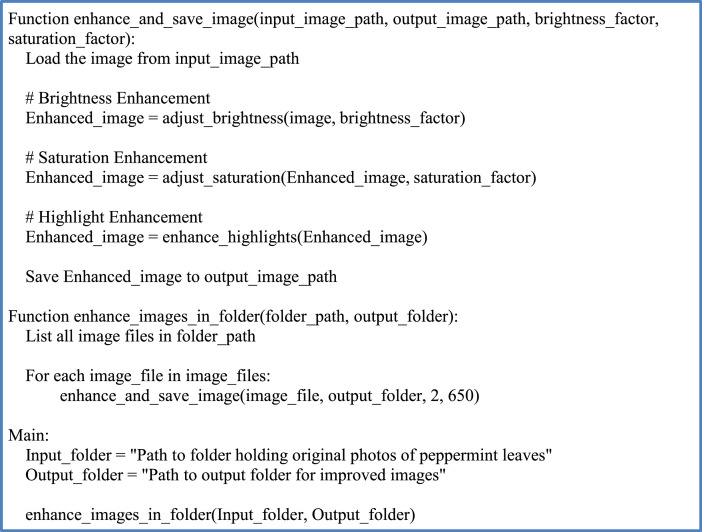
Pseudocode of image enhancement process.

**Fig. 5:**
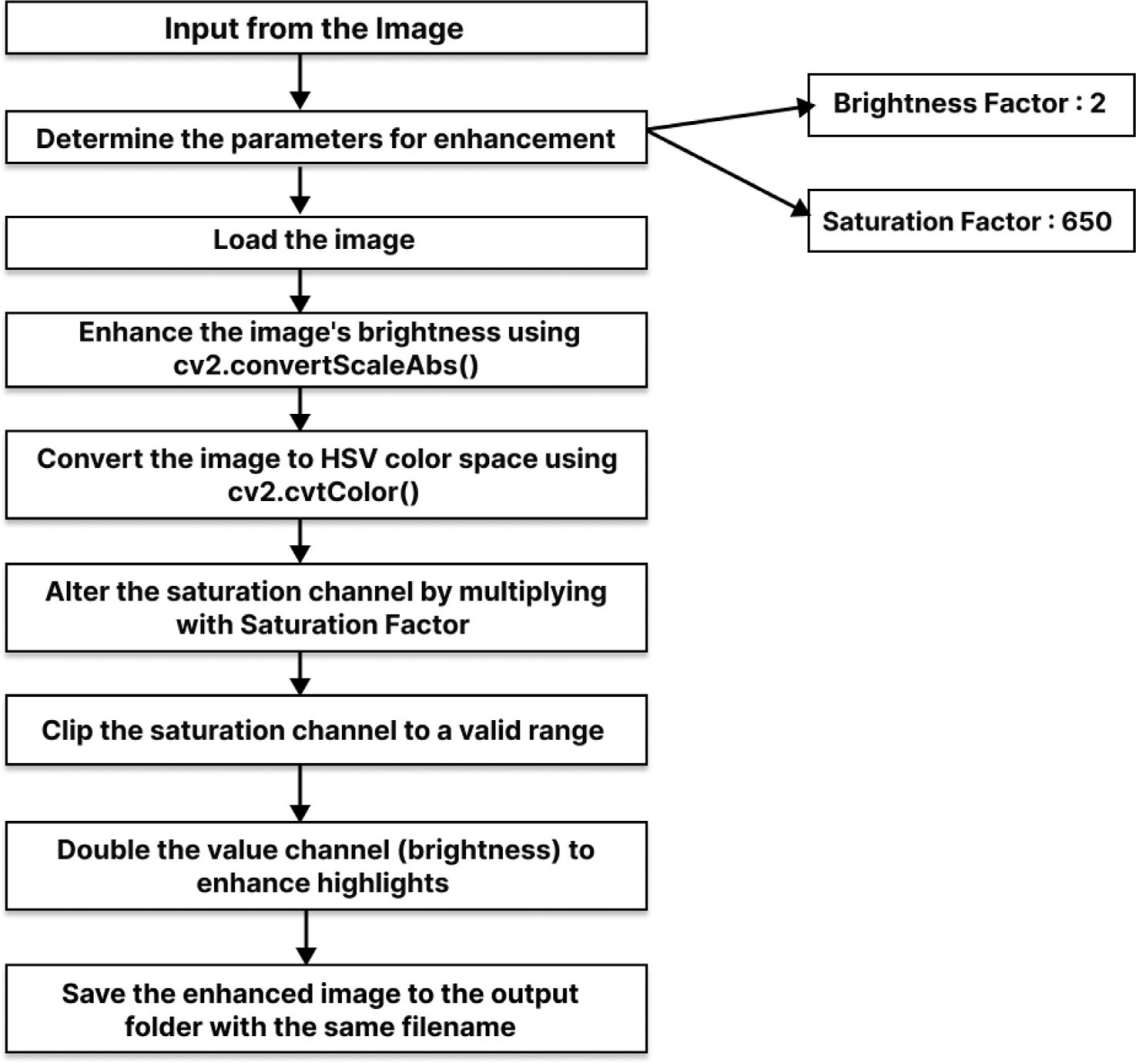
Flowchart of image enhancement steps.

A method for enhancing images of mint leaves is shown in Fig. 4, Fig. 5 shows flowchart of image enhancement steps. The method first adjusts the brightness and saturation of the image, and then enhances the highlights. The parameters for the brightness, saturation, and highlight enhancement are adjusted based on the lighting conditions of the original image. The original format of the images is now accessible to the public through Mendeley [7].

## 4 Evaluation of Machine Learning Models on the Mint Leaves Dataset

To demonstrate mint leaves dataset has the potential to be used to address a variety of research questions in the field of leaf quality assessment such as how machine learning algorithms be used to accurately discern the condition of mint leaves?, we evaluated the performance of three machine learning models on the dataset: VGG16, ResNet50, and MobileNetV2. These models are renowned for their capabilities in image recognition tasks. We pre-processed the dataset by resizing the images to 224×224 pixels, normalizing the pixel values, and randomly cropping and flipping the images. We also performed data augmentation by randomly rotating, translating, and shearing the images. This was done to artificially increase the size of the dataset and to prevent the models from overfitting. We fine-tuned the models using the Adam optimizer with a learning rate of 0.0001. The models were trained for 100 epochs. The results showed that all three models were able to achieve high accuracy on the training data. VGG16 achieved an accuracy of 88%, ResNet50 achieved an accuracy of 94%, and MobileNetV2 achieved an accuracy of 92%. These results indicate that the mint leaves dataset is valuable to researchers who are using machine learning models to perform condition or classification of mint leaves [8,9]. Table 2 summarizes the accuracy of the three models before and after training.

**Table 2:** Machine learning models accuracy on mint leaves dataset.

Model	Accuracy Before Training	Accuracy After Training on Mint Leaves dataset [7]
VGG16	0.2%	88%
ResNet50	0.4%	94%
MobileNetV2	0.25%	92%

## Limitation

Expanding the dataset to encompass a wider range of classes and samples from diverse global regions would enhance its overall diversity and applicability.

## Ethics Statement

Our study does not involve studies with animals or humans. Therefore, we confirm that our research strictly adheres to the guidelines for authors provided by Data in in terms of ethical considerations.

## Acknowledgments

We are grateful to Kasetsart University Sriracha Campus, Thailand and Vishwakarma University, Pune for their support and provision of necessary resources during this research endeavour.

## CRediT authorship contribution statement

**Rohini Jadhav:** Conceptualization. **Yogesh Suryawanshi:** Conceptualization, Writing – review & editing. **Yashashree Bedmutha:** Methodology, Data curation, Writing – original draft. **Kailas Patil:** Conceptualization, Supervision, Writing – review & editing. **Prawit Chumchu:** Writing – review & editing.

## Declaration of Competing Interest

The authors declare that they have no known competing financial interests or personal relationships that could have appeared to influence the work reported in this paper.

## Data Availability

Pudina Leaf Dataset: Freshness Analysis (Original data) (Mendeley Data)
